# Application of an Adsorption Process on Selected Materials, Including Waste, as a Barrier to the Pesticide Penetration into the Environment

**DOI:** 10.3390/ma15134680

**Published:** 2022-07-04

**Authors:** Jacek Piekarski, Katarzyna Ignatowicz, Tomasz Dąbrowski

**Affiliations:** 1 Faculty of Civil Engineering, Environmental and Geodetic Sciences, Koszalin University of Technology, 75-453 Koszalin, Poland; jacek.piekarski@tu.koszalin.pl (J.P.); tomasz.dabrowski@tu.koszalin.pl (T.D.); 2 Faculty of Civil Engineering and Environmental Sciences, Bialystok University of Technology, 15-351 Bialystok, Poland

**Keywords:** adsorption, aldrin, pesticide burials, compost from sewage sludge

## Abstract

The article presents research on using the adsorption process of aldrin (a chloro-organic pesticide that most often occurs in the environment near expired pesticide burials). The research used three sorbents: two activated carbons and compost from sewage sludge as a low-cost sorbent. Obtained adsorption isotherms belong to the L group according to the Giles classification. The test results and their analysis confirm that the IZO application facilitates the analysis of the adsorption process. The study results also confirm that compost can be a cost-effective alternative to commercial activated carbons to build barriers protecting the environment against existing leaking expired pesticide burials.

## 1. Introduction

There is still an unsolved problem of expired pesticides in many countries, especially those used in the 1970s [1,2]. These are dangerous, often mutagenic and carcinogenic compounds that accumulate in the human organism. The structure of the existing expired pesticide burials (EPBs) is deteriorating each year, which causes a significant threat of contamination to the natural environment. It poses a danger to residents because pollutants leak into the environment in imperceptible amounts. Groundwater, surface water, and soil become contaminated due to the infiltration of pesticide waste from leaky burials [3,4,5,6,7]. When reaching the aquifer, the leaks move along the direction of groundwater flow and may be transferred to the surface water.

For this reason, EPBs should be liquidated reliably and following the current knowledge. A constant inflow of pollutants into waters from corroded and damaged burial structures will continue for many years. It is worth remembering that even after the liquidation of an EPB, the effects of toxic substances will be observed for many years, both in soil and water. Hence, there is a need to look for methods to reduce pesticide migration in the environment and implement new solutions based on the adsorption process [4,5,8,9,10]. Several studies show that composts produced from organic materials, including sewage sludge, can be effective sorbents [11]. They were used on a laboratory scale to remove heavy metals [12,13,14], dyes [15], or plant protection chemicals [16,17,18]. However, the effectiveness of composts, especially in removing plant protection chemicals, may differ as it depends on many factors, including the structure and affinity of individual active ingredients to humic substances contained in the composts [11].

The adsorption process may be analyzed in various aspects, including kinetics, statics, and dynamics equations. Kinetics uses the equations of the adsorbate flow outside the adsorbent particle and the saturation of the adsorbent particles. The statics describes the last stage of adsorption. It concerns the state of equilibrium when the solute concentration in the solution is in equilibrium with the concentration of this substance on the adsorbent surface. The adsorbate separates between the solution and adsorbent phases in such a situation. The most frequently described method of describing such a separation is the dependence of the adsorbed substance amount per unit mass of the adsorbent as a function of the substance concentration in solution at a given temperature. In the mathematical notation, the equations of adsorption isotherms according to Freundlich, Langmuir, and BET are used most often. In turn, the adsorption dynamics is a combination of statics, kinetics, and the mass balance equations of the adsorbate. Different types of adsorption isotherms apply to different theories of adsorption [19,20,21,22,23,24].

Freundlich’s theory describes the monolayer adsorption phenomenon in which an experimental isotherm equation ${a=x}_{m}\cdot C_{r}^{1/n}$ is used, where *x_m_* expresses the adsorption capacity. On the other hand, the adsorption intensity of the substance is determined by the parameter *n.* Its increase means that adsorption is more intense. In the case of low concentration values, the Freundlich isotherm becomes a linear equation (*n* = 1). According to Freundlich’s formula, the amount of adsorbed substance may increase indefinitely for high values of equilibrium concentrations as they increase. In such conditions, the adsorbent surface is saturated. Therefore, the Freundlich isotherm is applicable for average equilibrium concentration values, coinciding with the Langmuir isotherm [20,24].

Langmuir’s theory assumes that the number of adsorbed particles cannot exceed the number of active sites when the adsorbent surface is covered. Then, the adsorption forces are isolated, which blocks the formation of subsequent layers, and an increase in the concentration value does not result in an increase in adsorption. The theory in mathematical notation assumes that the adsorbed layer is in dynamic equilibrium with the concentration in the solution, absence of interactions, and lack of ability to generate a multilayer. The theory also assumes the constant energy of adsorption; i.e., the surface is energetically homogeneous. The mathematical formula is as follows: ${a=x}_{m}\cdot \left[\left(k_{L}\cdot C_{r}\right)/\left({1+k}_{L}\cdot C_{r}\right)\right]$. The adsorption capacity at low and high equilibrium concentrations is linear. For low values, it is proportional to the equilibrium concentration. In contrast, it is parallel to the ordinate axis for high values, proving that the surface has reached the state of saturation [20,24].

Stephan Brunauer, Paul Emmett, and Edward Teller described the total amount of adsorbed substance per unit mass of adsorbent in the mathematical notation by the following formula: ${a=x}_{m}\cdot \left\{\left(k_{B}\cdot C_{r}\right)/\left[\left({1-C}_{r}\right)\left(1+\left(k_{B}-1\right)\cdot C_{r}\right)\right]\right\}$. BET theory is a development of the Langmuir adsorption model, introducing the possibility of molecule interactions. When the adsorbate molecule hits the occupied spot on the adsorbent surface, it creates adsorption complexes. In addition, as the solute concentration increases, the number of unoccupied active sites decreases, and adsorbate molecules may create further adsorption layers [20,24].

The adsorption process duration may be calculated using the adsorbate concentration field in the internal structure of the adsorbent model (batch systems) or adsorbate temporospatial distribution in the adsorbent bed model. When modeling the adsorption process on granular active carbon, the following simplifications are used: medium incompressibility, constant volume flow, homogeneous bed, constant porosity in each cross-section, constant mass transfer coefficient value, constant adsorption rate in the whole bed, and hydraulic flow diffusion causing the transport of the adsorbate [14,16].

The article presents research on using the adsorption process on selected materials, including waste materials (compost from sewage sludge), as a barrier preventing pesticides from inflowing into the environment and limiting their migration from the existing EPBs and warehouses. Despite numerous reports on the application of sewage sludge compost as a sorbent for pesticide removal from the soil, few studies discuss the problem of EPBs, their threat to the natural environment, and prevention methods. Therefore, the main aim of the conducted studies was to assess if compost from sewage sludge may be used as a sorbent preventing leaching pesticides into the soil environment and how it performs compared with commercial activated carbon sorbents. The compost is comparatively cheap and available material. The other aim of the studies was to verify the correctness of linearization of adsorption isotherms implemented in the IZO application by nonlinear estimation. IZO is the application developed by authors to analyze the adsorption process. In an accessible way, this program covers in detail the issues of calculating the duration of the adsorption process, using the value of the adsorbate concentration in the adsorbent layer obtained from adsorption isotherms [24].

## 2. Materials and Methods

(a) Sorbate

Based on literature data and studies, the authors selected chloro-organic pesticides that most often occur in drinking water near EPBs at the highest concentration as representative sorbate [1,2,6,25,26]. Individual pure active substance aldrin was applied. Technical grade aldrin of 99.8 ± 0.2% purity obtained from the Institute of Industrial Organic Chemistry Analytical Department in Poland was used as sorbate. A sample pesticide solution was prepared by dissolving 1 g of pesticide in 10 cm^3^ of acetone and then diluted to 1 dm^3^ with doubly distilled deionized water.

(b) Sorbent

**Figure 1:**
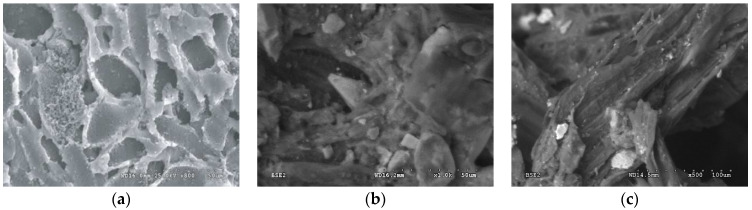
Scanning electron microscope (SEM) photos of NP-5 (**a**), WG-12 activated carbon (**b**), and compost (**c**).

The research used three sorbents: typical activated carbon produced from hard coal, activated carbon produced from waste coconut shells, and compost as an alternative waste sorbent. The microporous activated carbons NP-5 and WG-12 manufactured by Gryfskand Co. Hajnówka, Poland, were used as sorbents. Activated carbon NP-5 is produced from coconut shells and has a high surface area and higher adsorption capacity than other granular carbons. WG-12 is currently the most common carbon sorbent used in water treatment plants. Compost from sewage sludge obtained directly from a sewage treatment plant in Sokółka was used as a natural, low-cost sorbent. Table 1 and Table 2 and Figure 1 present characteristics of the sorbents used during investigations.

SEM images of the absorbents show that sewage sludge adsorbent (Figure 1c) is relatively flat, compact, and has fewer pores (compared to NP-5–Figure 1c). On the other hand, there is no significant difference in structure and pore number when comparing compost with WG-12 carbon (Figure 1b). The SEM images demonstrate that sewage sludge compost is less porous and more uniform with various, rather big, irregular fragments, on which there are a few particles, most likely of mineral origin.

The mechanical–biological wastewater treatment plant in Sokółka processes municipal and industrial (mainly dairy) wastewater. Its biological section uses SBR-type reactors. The plant’s capacity is 6000 m^3^/day, and the amount of sewage sludge produced is about 330 tons of d.m./year. The sewage treatment plant in Sokółka is a rare facility in the Podlasie region. The sludge is composted at the plant. Dewatered sludge is mixed with carbon carriers and sawdust and then is piled into prisms in special halls. Aerating grates are incorporated into the prism to remove gases that form during the composting. After the first composting phase, prisms are covered with a foil for the next three weeks. During that period, intensive sludge fermentation occurs, and the temperature increases up to 60 °C. Prisms are aerated during the next stage to create an aerobic environment (aeration time—approx. 14–20 days). Produced compost is kept in prisms for about 2.5 months [27]. This product was subjected to comprehensive tests at the Institute of Soil Science and Plant Cultivation Puławy, Poland, and a permit was obtained from the Polish Minister of Agriculture and Rural Development for producing and marketing an organic fertilizer under the name “Sokólski Compost”. It does not leach any toxic substances or heavy metals. It is approved for use in agricultural and horticultural crops. Thus, it might be used as a protective barrier for the environment in the vicinity of burial grounds.

(c) Analytical procedure

Aldrin concentrations were determined in collected samples according to obligatory methodology using an Agilent gas chromatograph equipped with ECD and NPD detector. The injector temperature was 210 °C, and the helium flow rate was 1.0 cm^3^/min. The column DB (35 m of length, 0.32 mm of ID, 0.5 μm film thickness) temperature was set at 120 °C for 2 min and increased at a rate of 13 °C/min to 190 °C. Finally, the temperature was raised to 295 °C and maintained isothermally for 20 min [20,28,29].

Validation based on the SANTE/11945/2015 transmitter was conducted to maintain the results’ credibility. During the validation process, the following parameters were determined: linearity, recovery, precision, limit of detection (LOD), limit of quantification (LOQ), matrix effect (ME), and uncertainty (U). The applied method ensured satisfying recovery (R) for all isomers within the 92–99% range. Precision calculated as relative standard deviation (RSD) was below 22%. The matrix effect did not substantially impact the decreasing or increasing signals for most compounds and was within the −13 and 12% range. Satisfactory linearity of the *R*^2^ > 0.999 correlation coefficient method was obtained in the analyzed concentration range. LOQ was at 0.1 µg/dm^3^, whereas LOD was at 0.03 µg/dm^3^. The extended uncertainty of measurements was, on average, between 8 and 22%.

(d) Sorption procedure

The studies under static conditions were conducted according to the American company Chemviron Carbon methodology and literature data [21,30,31,32,33,34,35,36]. They aimed to plot adsorption isotherms, making it possible to determine the sorption mechanism and sorption capacity under given conditions. The sorbent was degassed, washed with distilled water, dried, crushed with a ball mortar, and dried in an electric dryer at 150 °C for 3 h to a constant mass. From the material thus obtained, samples of 0.002, 0.004, 0.006, 0.010, 0.015, and 0.025 g were prepared, each for 100 cm^3^ of solution. The prepared sorbent samples were added to the 5 mg/dm^3^ pesticide solution into conical flasks. The vessels were shaken on a shaker at a constant vibration amplitude for 24 h, leaving them for 48 h to obtain complete sorption equilibrium. After this time, the samples were subjected to double filtration using soft tissue filters. The first and last portions of the filtrate were discarded. The concentration of contaminants in the filtrate was then determined according to the current methodology. Finally, the sorption process was analyzed using the IZO application based on the obtained result.

(e) Computer calculations

The IZO application linearizes adsorption isotherms. Freundlich isotherm equation *a = x_m_∙C_r_^n^* is transformed to *ln*(*a*) = *ln*(*b*_1_) + *b*_2_ ∙ *ln*(*C_r_*), where *x_m_* = *exp*(*b*_1_) and *n = b*_2_. Langmuir isotherm equation *a* = *x_m_* ∙ [(*K_L_* ∙ *C_r_*) ∙ (1 + *K_L_* ∙ *C_r_*)^−1^] is transformed to *a*^−1^ = *b*_1_ + *b*_2_ ∙ *C_r_*^−1^, where *x_m_* = *b*_1_^−1^, and *K_L_* = *b*_1_ ∙ *b*_2_^−1^. The application transforms BET isotherm equation *a* = *x_m_* ∙ (*K_B_* ∙ *C_r_*) ∙ [(1 − *C_r_*) (1 + (*K_B_* − 1) *C_r_*)]^−1^ to *C_r_* ∙ [*a* ∙ (1 − *C_r_*)]^−1^ = *b*_1_ + *b*_2_ ∙ *C_r_*, where *x_m_* = (*b*_1_ + *b*_2_)^−1^ and *K_B_* = 1 + *b*_2_ ∙ *b*_1_^−1^. Linear least-squares approximation is used to calculate coefficients *b*_1_ and *b*_2_. Then IZO calculates the coefficient of determination *R*^2^ and the regression’s standard error of the regression *S*. The *R*^2^ value (0 to 1) describes if the fitted curve explains the data variability correctly. *R*^2^ of 1 shows that the regression fits the data perfectly. The application presents lower and upper confidence limits and prediction intervals that determine the areas of probability value of 95% for a fitted plot or individual data points [22,24].

(f) Adsorption bed working time

The IZO application may also calculate the duration of the adsorption process *t_S_* (h). It uses three equations from literature. The value of *x_m_* (g/kg) (adsorbate concentration in the adsorbent monolayer) calculated in the first module is a primary parameter in those equations [22,24].

The first equation is a transformed mass balance formula (MBE) for the adsorbent layer:

(1) $$t_{S}=\frac{x_{m}\cdot g_{w}\cdot H}{v_{p}\cdot \left(C_{0}-C_{e}\right)},\;$$

where *H* (m) is a bed height, *g_w_* (kg/m^3^) is bulk density, *C*_0_ (kg/m^3^) is the initial concentration of the medium, *C_e_* (kg/m^3^) is the assumed output concentration, and *v_p_* (m/h) is flow velocity.

The ZZT (Zuchowicki, Zabieziński, and Tichonow) equation results from the transformation of the Langmuir equation:

(2) $$t_{S}=\frac{x_{m}\cdot g_{w}}{v_{p}\cdot C_{0}}\left\{{H-v}_{p}\cdot k_{e}^{-1}\left[w^{-1}\cdot \ln\left(C_{0}\cdot C_{e}-1\right)+\ln\left(C_{0}\cdot C_{e}\right)-1\right]\right\},$$

where *k_e_* (s^−1^) is the external mass transfer coefficient (obtained with *D* (m^2^/s), the averaged diffusion coefficient; *ε_W_* (-), the layer porosity; and *d* (mm), the average adsorbent grain sizes), *w* (-) is the quotient of *C*_0_ to *C*_0.5_ (adsorbate concentration in the stream equivalent to half the limit value of adsorption). A simplified version of the ZZT equation is easier to use:

(3) $$t_{S}=\frac{x_{m}\cdot g_{w}}{v_{P}\cdot C_{0}}\left\{{H-v}_{p}\cdot k_{e}^{-1}\left[\ln\left(C_{0}\cdot C_{e}\right)-1\right]\right\}.\;$$

The BA (Bohart–Adams) equation is similar to the ZZT Equation (2). However, it lacks *w* (-), so Equations (3) and (4) use only *x_m_* to refer to the adsorption isotherm.

(4) $$t_{S}=\frac{x_{m}\cdot g_{w}}{v_{p}\cdot C_{0}}\left\{{H-v}_{p}\cdot k_{e}^{-1}\left[\ln\left(C_{0}\cdot C_{e}-1\right)+\ln\left(C_{0}\cdot C_{e}\right)-1\right]\right\}.$$

The most significant impact on the *t_S_* value in Equations (2)–(4) is exerted by the *x_m_·g_w_*·(*v_p_·C*_0_)^−1^ part. A second part corresponding to bed height and the adsorption front has a lower impact [22,24].

The IZO application produces graphs of *t_S_* vs. the adsorption bed height *H* using Equations (1), (3), and (4). Usually, adsorption process duration *t_S_* calculated using Equation (1) is longer than that in actual conditions. Therefore, Zuchowicki, Zabieziński, and Tichonow (ZZT) or Bohart–Adams (BA) equations are used more often [22,24].

The IZO application allows changing other independent parameters and quickly calculates the *t_S_* time based on changes in those parameters [22,24].

## 3. Results and Discussion

Table 3 presents the results of the aldrin static adsorption process on the analyzed adsorbents.

Values given in Table 3 were introduced as initial data to the IZO application. The program calculated the values of coefficients of the linear and the classical equations of adsorption isotherms, according to Freundlich, Langmuir, and BET. The IZO app also calculated the quality measures of the model fit: the *R*^2^ coefficient and standard regression error *S* (Table 4).

**Figure 2:**
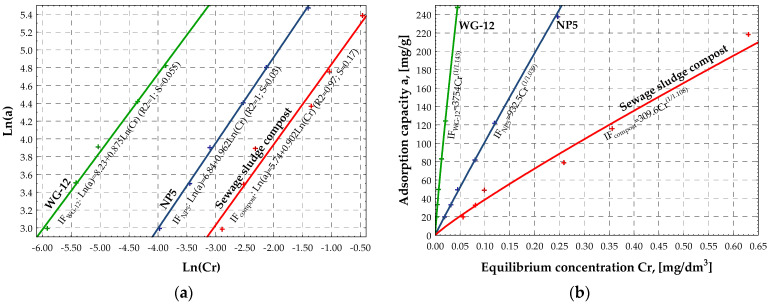
Graphs of linear isotherms (**a**) and classic isotherms (**b**) according to Freundlich for tested sorbents.

**Figure 3:**
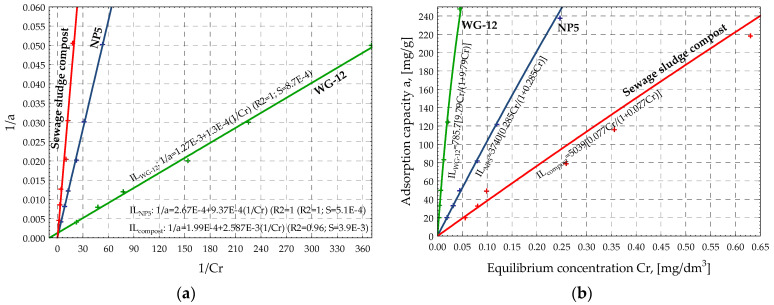
Graphs of linear isotherms (**a**) and classic isotherms (**b**) according to Langmuir for tested sorbents.

**Figure 4:**
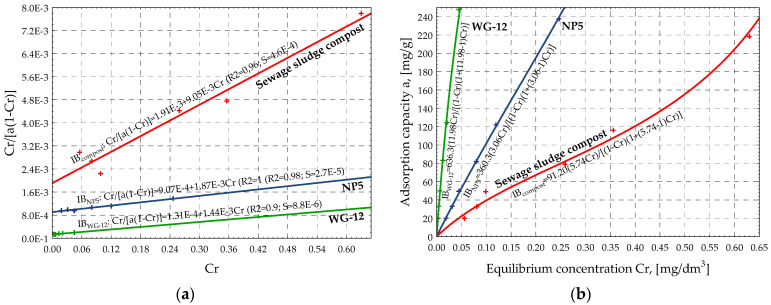
Graphs of linear isotherms (**a**) and classic isotherms (**b**) according to BET for tested sorbents.

Next, based on the values in Table 4, the IZO program generated linear graphs (Figure 2a, Figure 3a, and Figure 4a) and collective graphs of the adsorption isotherms (Figure 2b, Figure 3b, and Figure 4b) of the NP5, WG-12 activated carbons, and compost from sewage sludge. Figure 2b, Figure 3b, and Figure 4b present the adsorption isotherms of the selected pesticide (aldrin) on the investigated adsorbents as a change in adsorption capacity vs. change in equilibrium concentration of the adsorbate in the aqueous solution. The shape and arrangement of the curves are related to the different courses of adsorption in the range of low concentrations, resulting from the competitive nature of the interaction of the solute and solvent with the adsorbate surface.

One group of isotherms was obtained according to the Giles classification: L. This group includes isotherms of systems in which there is no high competitive interaction of solvent particles when the adsorbate populates active sites on the surface. In such a case, the planar arrangement of the aromatic ring of particles fills functional areas of the adsorbent.

The adsorption process of aldrin on NP5, WG-12 activated carbons, and compost is similarly described by the Freundlich, Langmuir, and BET isotherms. This conclusion is based on the graphs shown in Figure 2, Figure 3 and Figure 4 and the values of the *R^2^* and *S* coefficients (Table 4). The investigated adsorbents are characterized by a heterogeneous surface, and reactions occur in monolayer during adsorption along with additional interactions. That results in the multilayer adsorption effect. It is especially noticeable in the case of compost (Figure 4b).

Based on the values of the Freundlich isotherms, the adsorption intensity of the pesticide on the investigated activated carbons and the compost is similar (ranges from 0.87 to 0.97). At the same time, the highest sorption capacity of the adsorbent at the equilibrium concentration was observed for WG-12 active carbon, for which the adsorbate concentration in the adsorbent monolayer, based on the BET isotherm, was approximately *x_m_* = 636.3 g/kg. Activated carbon NP5 has a lower sorption capacity (*x_m_* = 360.3 g/kg), and compost from sewage sludge has the lowest (*x_m_* = 91.20 g/kg). The SEM demonstrates that sewage sludge compost is less porous and more uniform with various, rather big, irregular fragments, on which there are a few particles, most likely of mineral origin. At this initial stage of investigations, authors assumed that compost is mainly characterized by macro- and mesopores, with a small number of micropores, which directly translates into the amount of pesticide sorption. The results show that organic waste materials, such as compost from sewage sludge, adsorb aldrin and increase its retention significantly, thus decreasing its leaching. Results confirm findings presented in other reports [16,17,18].

Under actual conditions, the concentration of aldrin in the outflow from EPB is about *C_0_* = 5 mg/m^3^, while the groundwater flow velocity is maximum *v_P_* = 10 m/day. Model analysis of a protective barrier (of the assumed height of *H* = 10 cm) made from waste material, i.e., sewage sludge compost (of bulk density of *g_w_* = 385 kg/m^3^), was performed in the IZO app.

**Figure 5:**
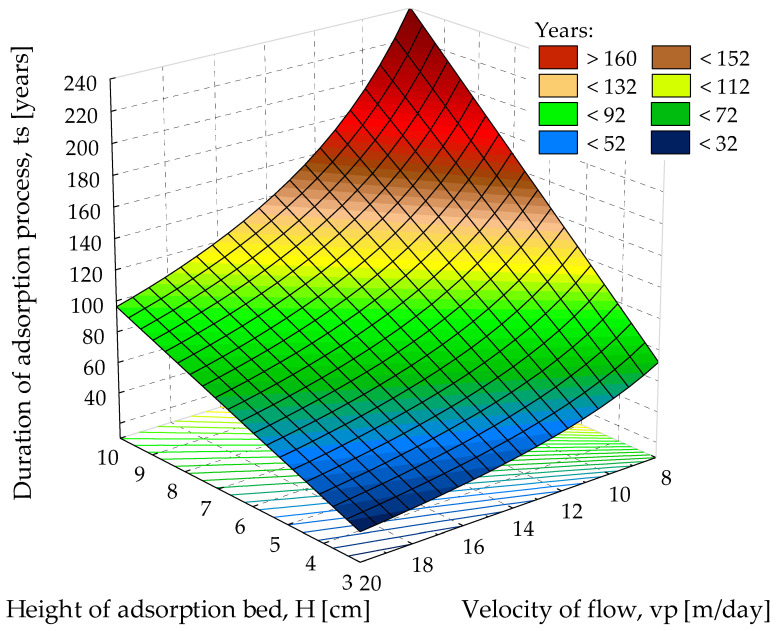
Graph of the working time of the sewage sludge compost bed *t_s_* (years) depending on bed height *H* (m) and flow velocity *v_P_*, based on results from the IZO app.

Obtained calculation results (based on Formula (1)) show that such a barrier ensures an actual duration of the adsorption process of about 200 years. Equations (3) and (4) cannot be used due to the value of *C_e_* being 0 mg/m^3^. However, assuming that the value of the final concentration is near 0 mg/m^3^, e.g., *C_e_* = 0.001 mg/m^3^, the duration of the adsorption process is also about 210 years (Figure 5). When NP5 or WG-12 activated carbons are used, the working time of such a barrier will relatively extend due to the higher values of the sorption capacity of those adsorbents. Thus, the application of the compost of sewage sludge may be an alternative solution, economically and technologically justified, to protect the environment surrounding the existing EPBs. Further kinetic studies and field-scale tests under actual conditions should be carried out to confirm the performance of sewage sludge compost and extrapolate lab-scale results.

## 4. Conclusions

The authors conducted model studies on using the adsorption process as a barrier to pesticide migration into the environment from the existing EPBs and storage facilities. Finally, the authors applied the IZO application to develop the results and analyze the adsorption process.

Obtained adsorption isotherms belong to the L group according to the Giles classification. It includes isotherms of systems where no strong competitive interaction of solvent particles occurs when the adsorbate populates active sites on the surface. The tested adsorbents are characterized by a heterogeneous surface. During the adsorption, not only monolayer reactions but also additional, distinct interactions occur. That results in multilayer adsorption, which is especially noticeable for compost.

The test results and analysis confirm that the IZO application facilitates studying the adsorption process. In particular, in a communicatively simple way, it calculates the coefficients and presents adsorption isotherms of Freundlich, Langmuir, and BET in the classical and linear system. Furthermore, the IZO application calculates the working time of the adsorption bed using formulas resulting from the transformation of the mass balance Equation (1), ZZT (3), and BA (4).

The study results confirm that the 10 cm thick barrier made of sewage sludge compost can protect the environment against aldrin for about 200 years (assuming that the concentration of aldrin in the leachate from EPB is 0.005 mg/dm^3^ and the velocity of groundwater flow is 10 m/day). Thus, compost can be considered a cost-effective alternative to commercial activated carbons to build barriers protecting the environment against existing EPBs.

## Figures and Tables

**Table 1:** Physical properties of activated carbon.

Parameter	Unit	WG-12	NP-5	Compost
Surface area	m^2^/g	900–1000	1300–1500	-
Total pore volume	cm^3^/g	0.8–0.9	min. of 0.7	-
Granulation	mm	1.2–2.0	0.75–1.2	-
Dechlorination	cm	5–7	5–8	-
Methylene blue	cm^3^	min. of 18	min. of 40	-
Iodine number	mg/g	850–950	1390	95
Hardness	%	90	95–97	-
Grindability	%	3.0	0.3	-

min. = minimum.

**Table 2:** Properties of compost.

Fertilizing components (mg/kg_dm_)						
Ca	Mg	Nog	N-NH_4_^+^	Pog	C	K
5.61	0.46	1.39	0.009	1.47	0.45	5.61
Metals content (mg/kg_dm_)						
Pb	Cu	Cd	Cr	Ni	Zn	Hg
7.0	22.7	0.63	9.9	5.8	210	2.5
Other (%)						
pH		Water content		Dry mass	Organic matter	
6.7		67.5		32.5	67.5	

**Table 3:** Influence of the adsorbent dose *m* (g) on the value of the equilibrium concentration *C_r_* (g/dm^3^) during the aldrin static adsorption process using NP5, WG-12 adsorbents, and sewage sludge compost.

Parameter	Sample No.					
	1	2	3	4	5	6
Initial concentration, *C*_0_ (mg/dm^3^)	5.00					
Adsorbent dose, *m* (g)	0.002	0.004	0.006	0.010	0.015	0.025
Equilibrium concentration, *C_r_* (mg/dm^3^)						
NP5 activated carbon	0.2466	0.1196	0.0801	0.0450	0.0317	0.0188
WG-12 activated carbon	0.0451	0.0210	0.0129	0.0065	0.0044	0.0027
Compost from sewage sludge	0.6300	0.3560	0.2590	0.0989	0.0807	0.0556

**Table 4:** Values of linear and classical coefficients of aldrin adsorption isotherms (Freundlich, Langmuir, and BET) for NP5, WG-12 activated carbons, sewage sludge compost, and the quality of the approximation.

Adsorbent	Isotherms	Linear Equation				Classical Equation	
		Coefficient	Value	*R^2^*	*S*	Coefficient	Value
NP-5	Freundlich	*a*	6.840	1.00	3.0 × 10^−2^	*k*	932.5
		*b*	0.962			*n*	1.039
	Langmuir	*a*	2.67 × 10^−4^	1.00	5.1 × 10^−5^	*x_m_*	3740
		*b*	9.37 × 10^−4^			*K_L_*	0.285
	BET	*a*	9.07 × 10^−4^	0.98	2.7 × 10^−6^	*x_m_*	360.3
		*b*	1.87 × 10^−3^			*K_B_*	3.06
WG-12	Freundlich	*a*	8.230	1.00	5.5 × 10^−2^	*k*	3754
		*b*	0.875			*n*	1.143
	Langmuir	*a*	1.27 × 10^−3^	1.00	8.7 × 10^−4^	*x_m_*	785.7
		*b*	1.30 × 10^−4^			*K_L_*	9.79
	BET	*a*	1.31 × 10^−4^	0.90	8.8 × 10^−6^	*x_m_*	636.3
		*b*	1.44 × 10^−3^			*K_B_*	11.98
sewage sludge compost	Freundlich	*a*	5.740	0.97	0.17	*k*	309.6
		*b*	0.902			*n*	1.108
	Langmuir	*a*	1.99 × 10^−4^	0.96	3.9 × 10^−3^	*x_m_*	5039
		*b*	2.59 × 10^−3^			*K_L_*	0.077
	BET	*a*	1.91 × 10^−3^	0.96	4.6 × 10^−4^	*x_m_*	91.20
		*b*	9.05 × 10^−3^			*K_B_*	5.737
